# In Situ Myocardial Regeneration With Tissue Engineered Cardiac Patch Using Spheroid-Based 3-Dimensional Tissue

**DOI:** 10.1016/j.atssr.2023.11.014

**Published:** 2023-11-30

**Authors:** Yojiro Koda, Tatsuya Watanabe, Keigo Kawaji, Fei Mo, Andrew D. Beaser, Marcella Vaicik, Narutoshi Hibino, Takeyoshi Ota

**Affiliations:** 1 Department of Surgery, University of Chicago Medicine, Chicago, Illinois;; 2 Department of Biomedical Engineering, Illinois Institute of Technology, Chicago, Illinois;; 3 Department of Medicine, University of Chicago Medicine, Chicago, Illinois

## Abstract

**BACKGROUND:**

We have developed a tissue engineered cardiac patch derived from a 3-dimensional (3D) myocardial tissue reinforced with extracellular matrix in an effort to enhance in situ myocardial regeneration. The feasibility of the patch was evaluated in a porcine model by various modalities to assess both the constructive and functional aspects of regeneration.

**METHODS:**

A spheroid-based 3D multicellular tissue was created using a 3D net mold system that incorporated cardiomyocytes and embryonic fibroblast cells. The 3D multicellular tissue was incorporated with extracellular matrix sheets and surgically implanted into the right ventricle of a healthy porcine model (n = 4). After 60 days, the implanted patches were evaluated by cardiac magnetic resonance imaging and electroanatomic mapping studies as well as by post-euthanasia analyses, including measurements of mechanical viscoelasticity.

**RESULTS:**

Cardiac magnetic resonance imaging revealed improved regional tissue perfusion in the patch area. Electroanatomic mapping exhibited regenerated electrical conductivity in the patch, as evidenced by relatively preserved voltage regions (1.11 ± 0.8 mV) in comparison to the normal right ventricle (4.7 ± 2.8 mV). Histologic and tissue analyses confirmed repopulation of site-specific host cells, including premature cardiomyocytes and active vasculogenesis. These findings were supported by quantitative reverse transcription–polymerase chain reaction.

**CONCLUSIONS:**

The tissue engineered cardiac patch effectively facilitated in situ constructive and functional myocardial regeneration, characterized by increased regional tissue perfusion and positive electrical activity in the porcine model.

Myocardial regenerative therapy employs bio-materials incorporating cellular components, such as stem cells, to foster tissue remodeling. In addition, stem cell–derived cardiomyocytes have been used to directly enhance myocardial function; however, challenges persist in cell viability and strength for blood pressure endurance without scaffolds. Whereas the extracellular matrix (ECM) scaffold demonstrates strength issues with the speed and direction of regeneration, attempts to promote regeneration through growth factors have yielded limited success.^1^ The application of spheroid-based tissue engineering offers a scaffold-free 3-dimensional (3D) tissue creation, capitalizing on increased cell density and improved paracrine effects.^2^

In this study, we have developed a tissue engineered cardiac patch from a spheroid-based 3D multicellular tissue to enhance in situ myocardial regeneration. The evaluation in a porcine model encompassed cardiovascular magnetic resonance (CMR), electroanatomic mapping, and mechanical property measurements.

## MATERIAL AND METHODS

### PREPARATION OF A SPHEROID-BASED 3D MULTICELLULAR TISSUE PATCH (MT PATCH).

**FIGURE 1:**
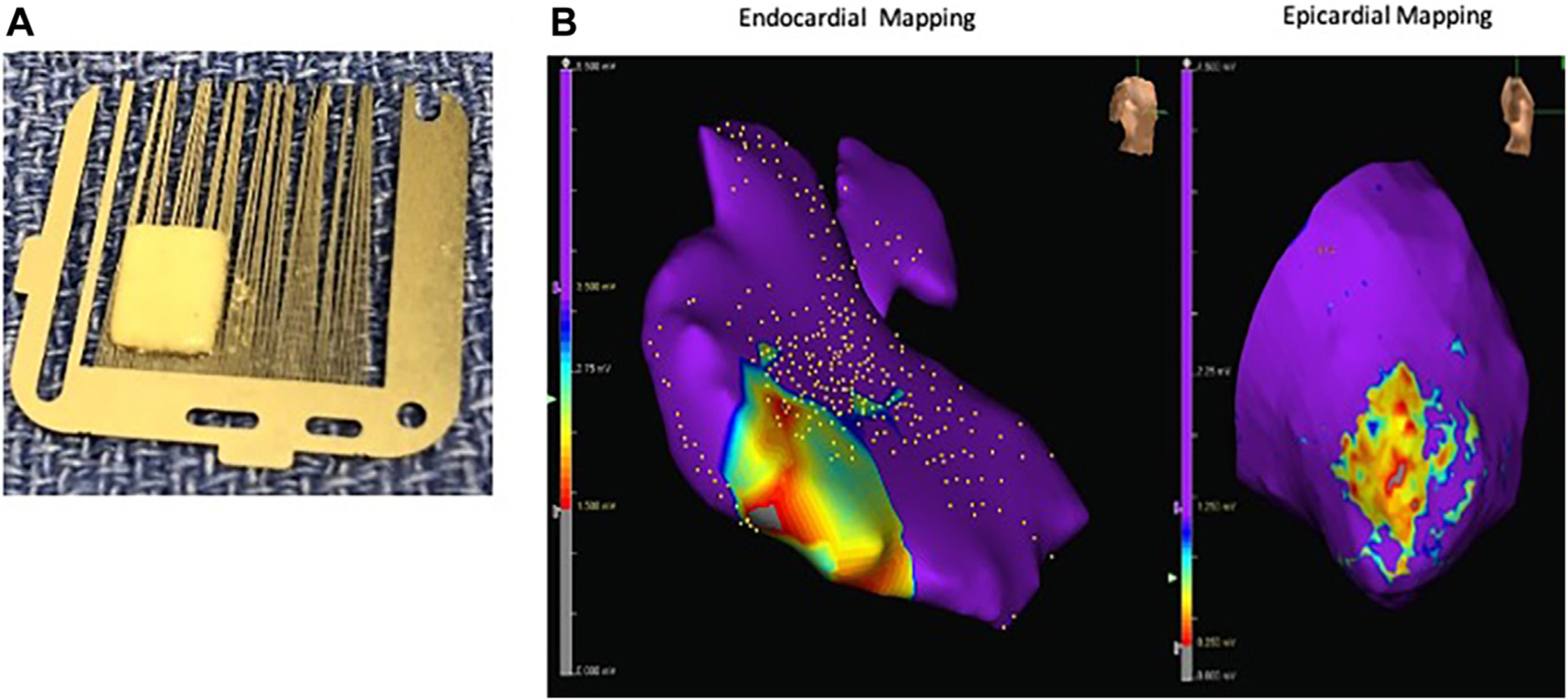
Spheroid-based 3-dimensional multicellular tissue. (A) Macroscopic view. (B) Epicardial and endocardial electroanatomic mapping of the multicellular tissue patch (MT patch) area. Left, endocardial mapping (purple: voltage, **>**3.5 mV; blue-gray in MT patch: voltages, 0–3.5 mV). Right, epicardial mapping (purple: voltage, **>**1.25 mV; blue-gray in MT patch: voltages, 0–1.25 mV).

H9c2 (ATCC #CRL-1446, cardiac myoblasts) and 3T3 (ATCC #CRL-1658, embryonic fibroblast) cells were combined at a ratio of 7:3, seeded into a microfabricated culture dish (EZSPHERE Cat#4000–905SP; Nacalai USA) at 33,000 cells in each well, and co-cultured in Dulbecco’s modified Eagle medium (DMEM)/Ham’s F-12 Nutrient Mixture (DMEM/F12; Thermo Fisher Scientific) supplemented with 10% fetal bovine serum, penicillin (100 IU/mL), and streptomycin (100 μg/mL). Microfabricated culture dishes were cultured in an incubator at 37 °C, 5% carbon dioxide, and 95% humidity for 72 hours to form cell spheroids. Collected spheroids (4000 spheroids) were seeded into a 3D net mold (TissueByNet) cavity of predetermined sizes for 14 days in a 6-well plate with DMEM/F12 with 10% fetal bovine serum, penicillin (100 IU/mL), and streptomycin (100 μg/mL) and cultured in an incubator at 37 °C, 5% carbon dioxide, and 95% humidity. Resultant cell blocks were removed from the 3D net mold system (Figure 1A). The 3D engineered multicellular tissues were then cultured in free-floating media to maturity, confirmed by staining with hematoxylin-eosin and 4′,6-diamidino-2-phenylindole (Vector Laboratories) and troponin T (Dako; Supplemental Figure 1).

ECM sheets derived from porcine small intestine submucosa (CorMatrix Cardiovascular) were trimmed and soaked in saline for 30 minutes at room temperature. The 3D multicellular tissue was embedded in the small intestine submucosa–ECM.

### VISCOELASTICITY.

After excision of the tissue in the patch area and left ventricle (LV) for a reference, the tissues underwent a 3-step decellularization process (10 mM Tris–1 mM EDTA, 0.5% sodium dodecyl sulfate, Dulbecco’s phosphate-buffered saline).^3^ Mechanical properties of the ECM (G′ elastic and G″ viscous) in decellularized samples were mechanically tested with an oscillating disc rheometer by employing a frequency sweep and constant shear strain (20%). A user-controlled compressive load was applied normal to the tissue samples at a constant 37 °C.

### SURGICAL IMPLANTATION OF AN MT PATCH.

The study protocol was approved by the Institutional Animal Care and Use Committee of the University of Chicago (#72513).

A female pig, mixed breed of Yorkshire and Landrace (20–30 kg; MT patch group, n = 4; control, n = 4), was anesthetized, and the heart was exposed through a right anterolateral thoracotomy. A tangential clamp was placed on the right ventricle (RV) free wall, which was incised in full thickness, and a 20-mm MT patch was implanted. At 60 days after implantation, the animals underwent CMR imaging and electromechanical mapping and were euthanized for tissue analyses. Daily oral cyclosporine (10 mg/kg) and methylprednisolone (1.6 mg/kg) were given from 2 days before patch implantation until euthanasia.

### CMR IMAGING.

**FIGURE 2:**
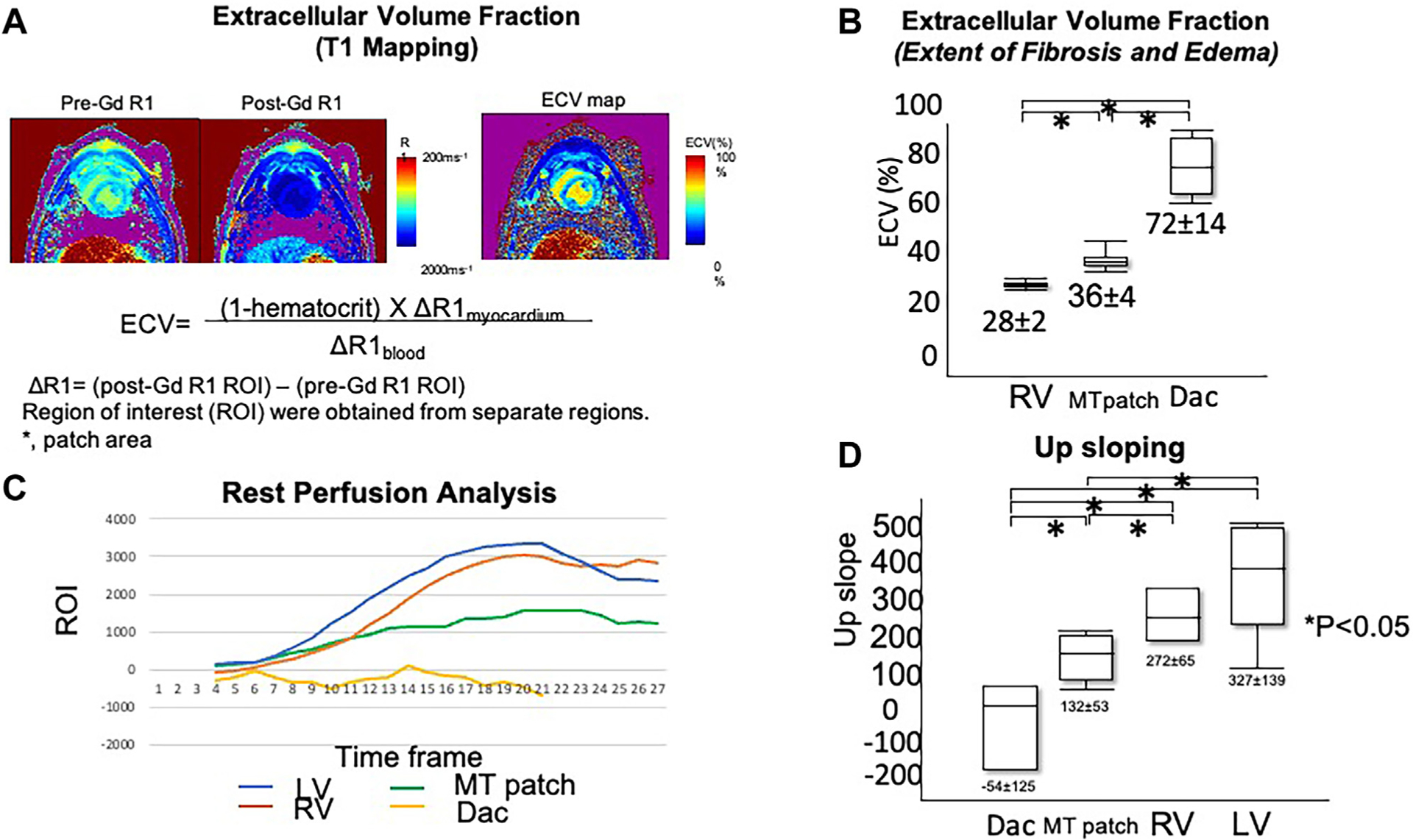
(A) Mechanism and equation for extracellular volume (ECV) fraction. (B) Extracellular volume fraction of each region. (C) Tissue perfusion with time frame. (D) The value of upsloping of perfusion. (Dac, Dacron patch; Gd, gadolinium; LV, left ventricle septum; MT patch, multicellular tissue patch; ROI, region of interest; RV, right ventricle.)

CMR assessment was performed after the 60-day period to evaluate the regional physiomechanical properties. All animals were imaged on 3T magnetic resonance imaging hardware (Philips Achieva) with a 28- to 32-channel torso array. In this CMR protocol, an initial localization scouts, followed by 2-, 3-, and 4-chamber views; short-axis cine CMR sequences are first acquired to determine the patch location, followed by the following sequences: (1) first-pass myocardial perfusion, (2) late gadolinium enhancement, and (3) T1 mapping (Figure 2). All imaging techniques were performed under respirator-controlled breath holding. The details were previously described.^1^

### EPICARDIAL-ENDOCARDIAL ELECTROANATOMIC MAPPING.

Under anesthesia, cutaneous mapping electrodes were attached to the EnSite Precision mapping system (Abbott Cardiovascular). A 7F or 8 F catheter (Livewire 2-2-2 duo-decapolar mapping catheter/Advisor HD Grid Mapping Catheter Sensor Enabled) was advanced into the RV through the right internal jugular vein or right femoral vein. The catheter was maneuvered throughout the porcine right ventricular endocardium, collecting voltage and geometric recordings (electroanatomic mapping) to generate a 3D model of the RV depicting electrical activity within the cardiac chamber. After endocardial mapping, epicardial mapping was similarly performed.

### HISTOLOGY.

The sections were stained with hematoxylineosin or prepared for immunohistochemical/fluorescence staining. Monoclonal antibodies specific for von Willbrand factor (vWF; Dako), tropomyosin (Santa Cruz Biotechnology), and a-sarcomeric actinin (Sigma) in pigs were used for immunohistochemical staining. The vWF-positive capillaries were counted under 200× microscopy. Twenty fields were randomly selected in each patch. Capillary density was expressed as the mean number of vessels per square millimeter.^4^

### QUANTITATIVE RT-PCR.

PrimeTime reverse transcription–polymerase chain reaction (RT-PCR) assays probe (Integrated DNA Technologies) and DNA Engine Opticon 2 (Bio-Rad Laboratories) were used, as previously described.^4^ The tissues were taken from the center of the patches and stored in an RNA stabilization reagent (RNA*later*; Qiagen). Analyzed transcripts included smooth muscle 22α, vimentin, β-myosin heavy chain, vWF, basic fibroblast growth factor, and vascular endothelial growth factor. Results were normalized to the level of porcine glyceraldehyde-3-phosphate dehydrogenase transcripts.

### STATISTICAL ANALYSIS.

Data were expressed as mean (SD). The statistical differences were determined by the Student *t*-test and Wilcoxon rank sum test with JMP software (SAS Institute).

## RESULTS

### VISCOELASTICITY.

Using an oscillating disc rheometer at an initial 0.1 rad/s angular frequency, patch area ECM shear storage modulus was 130 ± 70 Pa and shear loss modulus was 65 ± 36 Pa, contrasting with LV reference values of 420 ± 220 Pa (storage) and 130 ± 66 Pa (loss). Both storage and loss modulus increased by 48% (patch) and 35% (LV reference) at 1 rad/s.

### CMR IMAGING.

#### Extracellular Volume Fraction.

The values of RV extracellular volume fraction were statistically lower (28.4 ± 2.4) than in the MT patch group (36.1 ± 3.6; *P* = .012) and the Dacron group (72.0 ± 13.6; *P* = .00023). The extracellular volume fraction values in the MT patch group were significantly lower than those in the Dacron group (*P* = .0027; Figures 2A, 2B), suggesting that the MT patch group exhibited less fibrosis tissue and more viable tissue.

#### Rest Perfusion Analysis.

Relative maximum upslope, obtained from signal intensity curve analysis (Figures 2C, 2D), was highest in the LV group, similar to the RV group (LV group, 327 ± 139; RV group, 272 ± 65; *P* = .60). Values of the LV group were significantly higher than in the MT patch group and Dacron patch group (MT patch group, 132 ± 53 [*P* = .031]; Dacron group, −54 ± 125 [*P* = .015]). MT patch values were significantly higher than in the Dacron group (*P* = .044), indicating regional perfusion in the patch area, less than in the native myocardium.

### ENDOCARDIAL-EPICARDIAL ELECTROANATOMIC MAPPING.

The operator was blinded to the patch location on endocardial mapping; 3852 to 8760 (mean, 5710 ± 2174) endocardial points were taken in 4 procedures and re-created right ventricular chambers in the interface. Combined mapping data revealed positive electrical conductivity in the patch areas with reduced but still present voltage regions relative to the normal RV (patch, 1.11 ± 0.8 mV; RV, 4.70 ± 2.8 mV; Figure 1B).

### HISTOLOGY.

**FIGURE 3:**
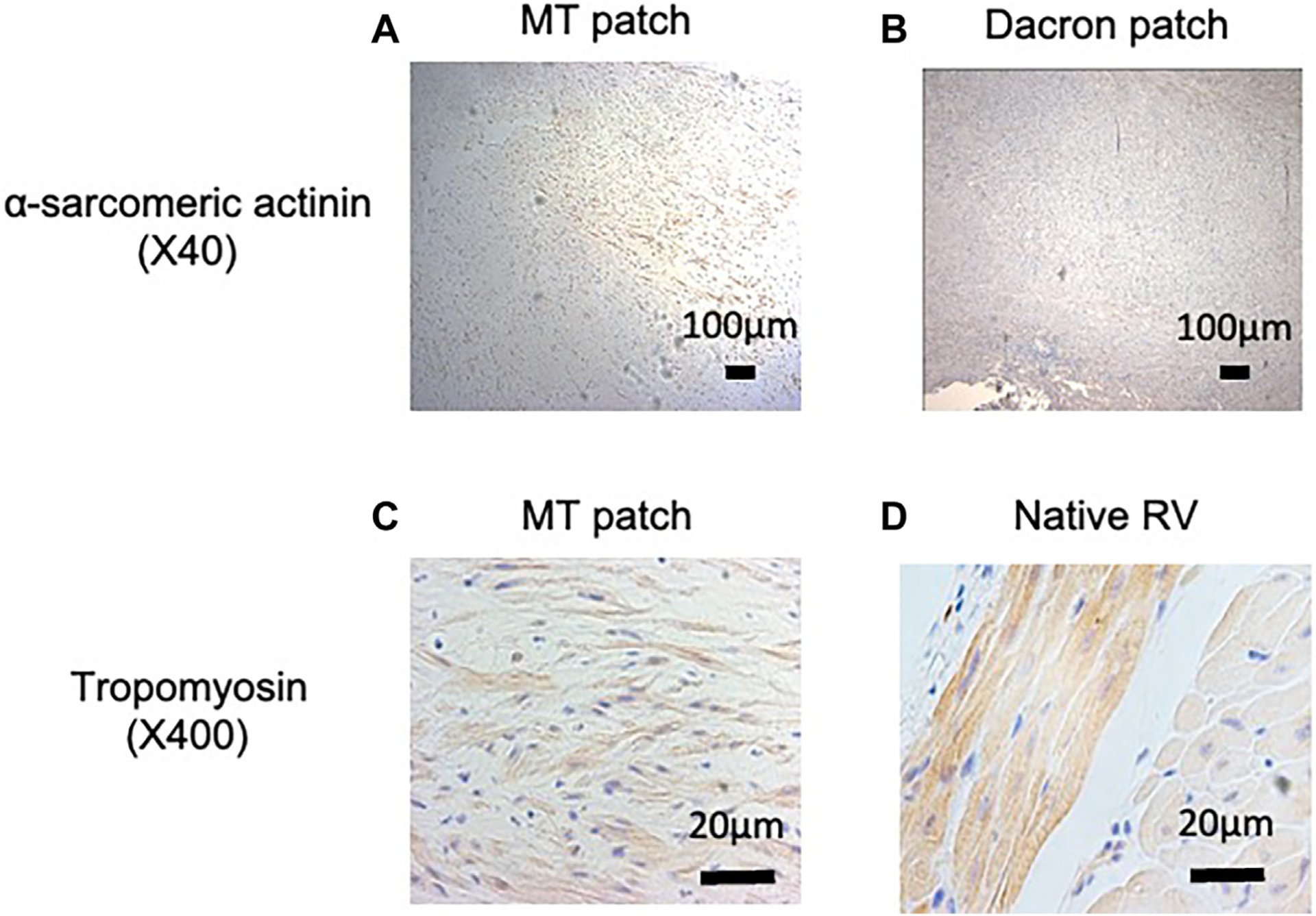
Histology and immunohistochemistry. (A, C) Scattered distribution of both tropomyosin- and a-sarcomeric actinin–positive cells in multicellular tissue (MT) patch. (B) No expression of α-sarcomeric actinin in Dacron. (D) Well-organized tropomyosin-positive cells in native right ventricle (RV).

Histology results are shown in Figure 3. Hematoxylin-eosin staining revealed repopulated cells in the MT patch, scattered tropomyosin, and α-sarcomeric actinin–positive cells, representing early and maturing cardiomyocytes.^5^ The surface of the patches was covered with a monolayer of endothelial cells (vWF positive).

Capillary density was significantly greater in the patch area than in the normal RV (patch, 14.7 ± 6.8/mm^2^; RV, 10.1 ± 4.0/mm^2^; *P* = .0004; Supplemental Figure 2).

### QUANTITATIVE RT-PCR.

**FIGURE 4:**
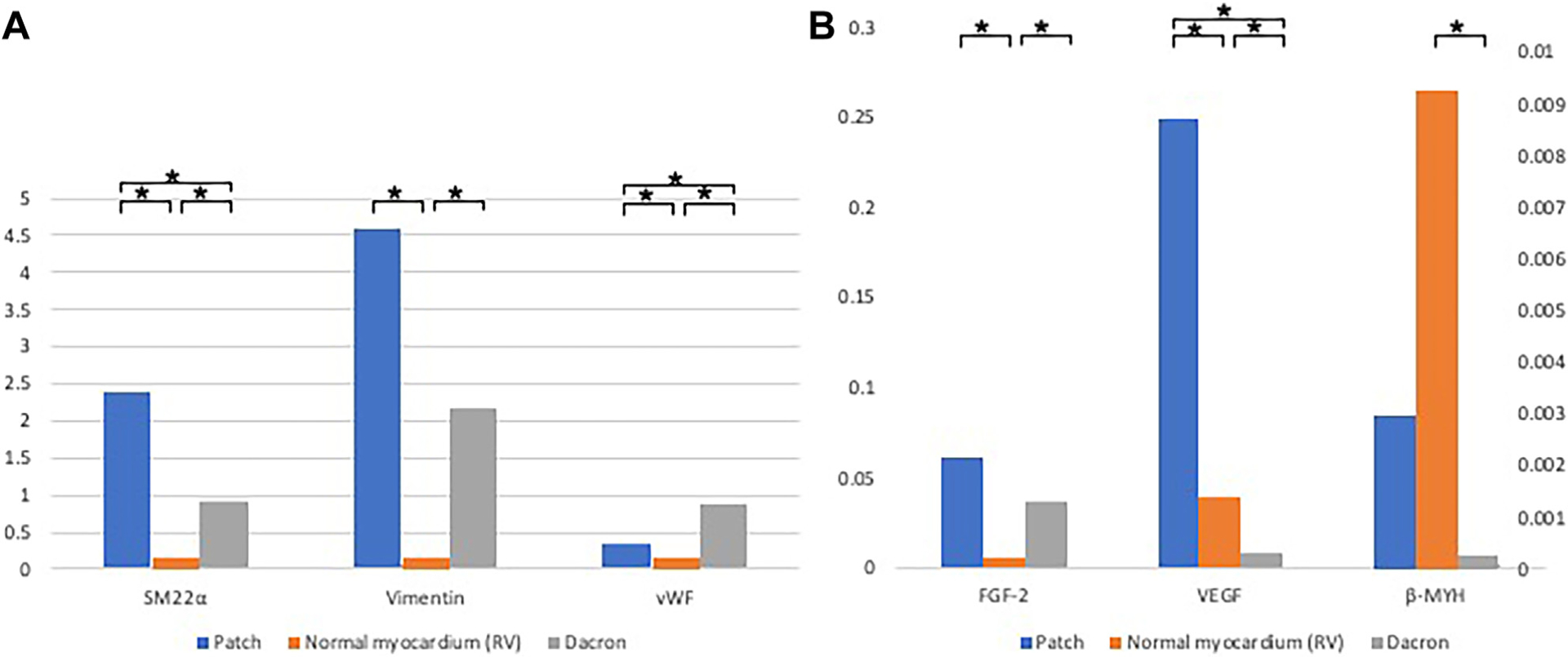
Reverse transcription–polymerase chain reaction (A) SM22a, Vimentin, vWF. (B) FGF-2, VEGF, beta-MYH. Messenger RNA expression was normalized with the value of glyceraldehyde-3-phosphate dehydrogenase expression. *P **<** .05. (β-MYH, β-myosin heavy chain; FGF-2, fibroblast growth factor 2; RV, right ventricle; SM22α, smooth muscle 22α; VEGF, vascular endothelial growth factor; vWF, von Willbrand factor.)

The results are summarized in Figure 4 and Supplemental Tables 1 and 2. Markers indicating premature mesenchymal cells (ie, smooth muscle 22α, vimentin) were expressed in the implanted cardiac patch more than in the healthy RV. The expression of vWF in the cardiac patch was statistically higher than in the normal myocardium (*P* = 2.7 × 10^−4^). The expressions of β-myosin heavy chain, a marker for the mature cardiomyocyte, in the MT patch did not reach the level of the healthy RV but was significantly higher than in the Dacron patch. Vascular endothelial growth factor was expressed more in the MT patch than in both the RV and Dacron patch. Fibroblast growth factor was significantly highly expressed in the MT patch and Dacron patch compared with the normal RV. The high expression of growth factors in the MT patch suggests remodeling involvement.

## COMMENT

The use of a spheroid-based 3D MT patch facilitated the proliferation of site-specific cells with the early signs of positive myocardial remodeling (Supplemental Figure 3). Histologic analysis confirmed the presence of vWF-positive endothelial cells homogeneously covering the endocardial side of the implanted patch as well as scattered tropomyosin- and α-sarcomeric actinin–positive cells, indicative of premature cardiomyocytes within the implanted MT patch. Overall, constructive repopulation of cells was evident throughout the entire layer of the implanted patch, accompanied by well-developed angiogenesis/vasculo-genesis, indicating active tissue blood perfusion. The implanted MT patch was found to be histologically in the early stages of myocardial regeneration, distinct from the tissue composition of a Dacron patch.

The results of RT-PCR supported the histologic findings. The strong expression of growth factors observed suggests that tissue regeneration is actively occurring, and the MT patch appears to be progressing toward successful myocardial regeneration. Furthermore, CMR contrast-enhanced perfusion suggests a positive effect of the implanted patch on functional remodeling. Electromechanical mapping revealed regenerated electrical conductivity in the patch, as evidenced by relatively preserved voltage regions compared with a healthy RV.

Viscoelasticity, which is often used as a value for the mechanical durability of a material against external forces, can be measured in myocardial tissue as well. In this study, viscoelasticity of the implanted MT patch area at the 60-day time point was restored to one-third of the healthy LV. It suggested that the in situ cyto-skeletal regeneration effect of the MT patch is promising.

In myocardial regeneration, it is essential to achieve histologic regeneration but also functional regeneration, including electrical conductivity. Our study aimed to quantitatively compare the electrical potentials between the patch area and surrounding normal myocardium. We demonstrated that the regeneration of cardiomyocytes is accompanied by at least partial reorganization of the electrical conduction system, indicating the occurrence of structural cardiac tissue regeneration.

Myocardial fibrosis has been recognized as a major contributor to arrhythmias in patients with myocardial infarction or cardiomyopathy. Our findings indicated that the MT patch area exhibited less fibrosis and reduced edematous change compared with the Dacron patch (ie, scarred tissue), and there were no arrhythmias or related death observed during the course of the project. We believe that our approach to promote constructive myocardial regeneration, including the electrical conductivity, has the potential to yield arrhythmogenic-free tissue.

### LIMITATIONS.

First, the RV model was used in this proof-of-concept study because it was cost-effective and to decrease mortality and morbidity in the porcine model. Future work should extend testing to left ventricular models, including a long-term model for a more comprehensive view. Second, the current results cannot determine their origin (host vs donor). A more rigorous analysis will be necessary.

### CONCLUSION.

The tissue engineered cardiac patch effectively facilitated in situ constructive and functional myocardial regeneration, characterized by increased regional tissue perfusion and positive electrical activity in the porcine model.

## Supplementary Material

1

## IN SHORT

- Spheroid-based multicellular patches facilitate in situ constructive and functional myocardial regeneration, such as increased regional perfusion and positive electrical activity.
- Combining extracellular matrix patch technology with cellular therapy has the potential to enhance physical strength and to accelerate the process of tissue regeneration.

## Abbreviations and Acronyms

3D: 3-dimensional
CMR: cardiovascular magnetic resonance
DMEM: Dulbecco’s modified Eagle medium
ECM: extracellular matrix
LV: left ventricle
MT patch: multicellular tissue patch
RT-PCR: reverse transcription–polymerase chain reaction
RV: right ventricle
vWF: von Willbrand factor
