# Supplementary material for: In Situ Myocardial Regeneration With Tissue Engineered Cardiac Patch Using Spheroid-Based 3-Dimensional Tissue
Source: Ann Thorac Surg Short Rep. Author manuscript; Available in PMC 2024 Mar 8. (PMC10922669; doi:10.1016/j.atssr.2023.11.014)
Supplement: 1 [file NIHMS1971550-supplement-1.docx]

Supplement


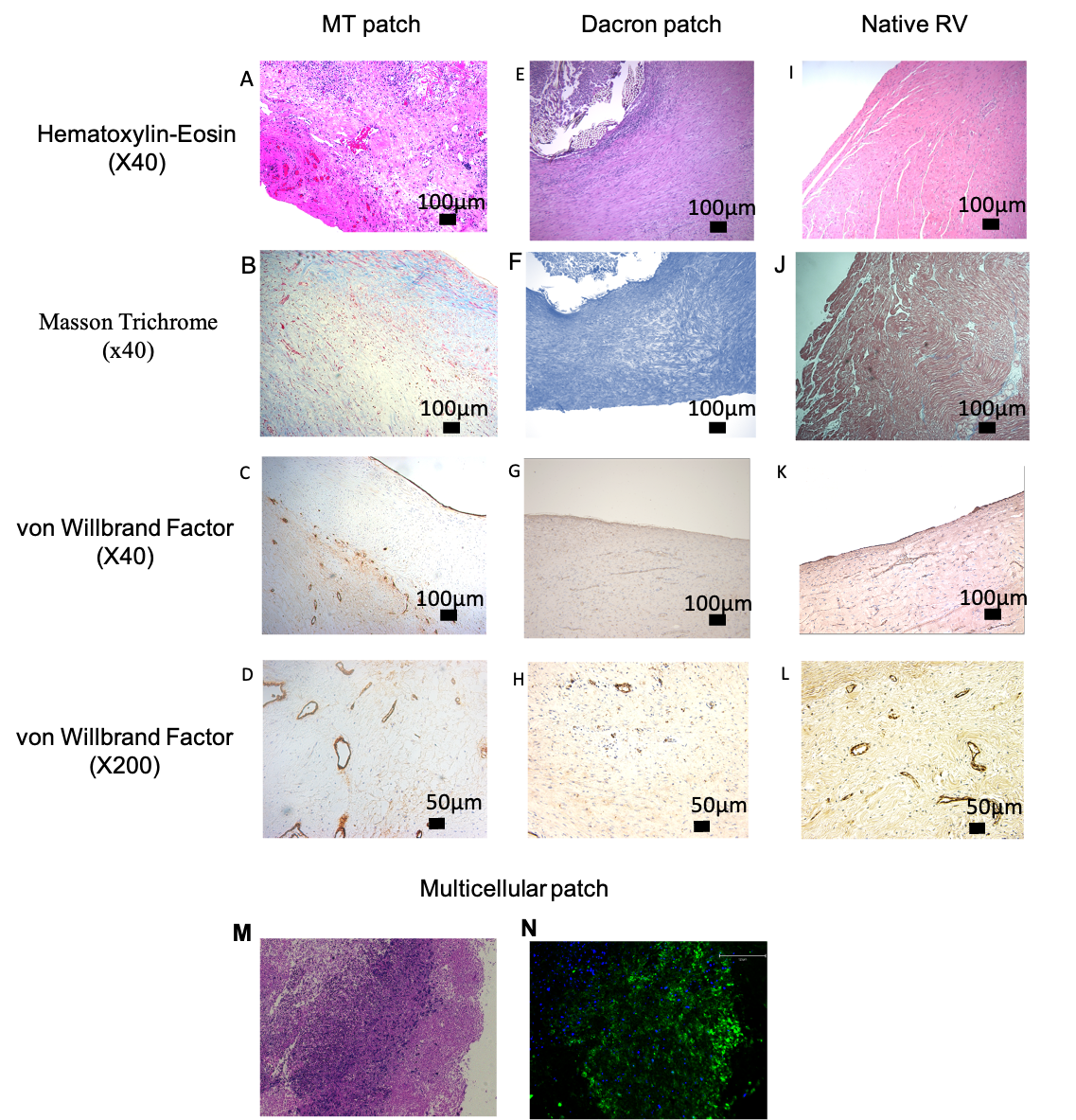


Supplement Figure 1: Histological analysis

A-D: MT patch, E-H: Dacron patch, I-L: Native Right ventricle, MN: Multicellular patch before implantation. Histological results MT patch showed less fibrous tissue, but distributed cell and high density of capillaries.


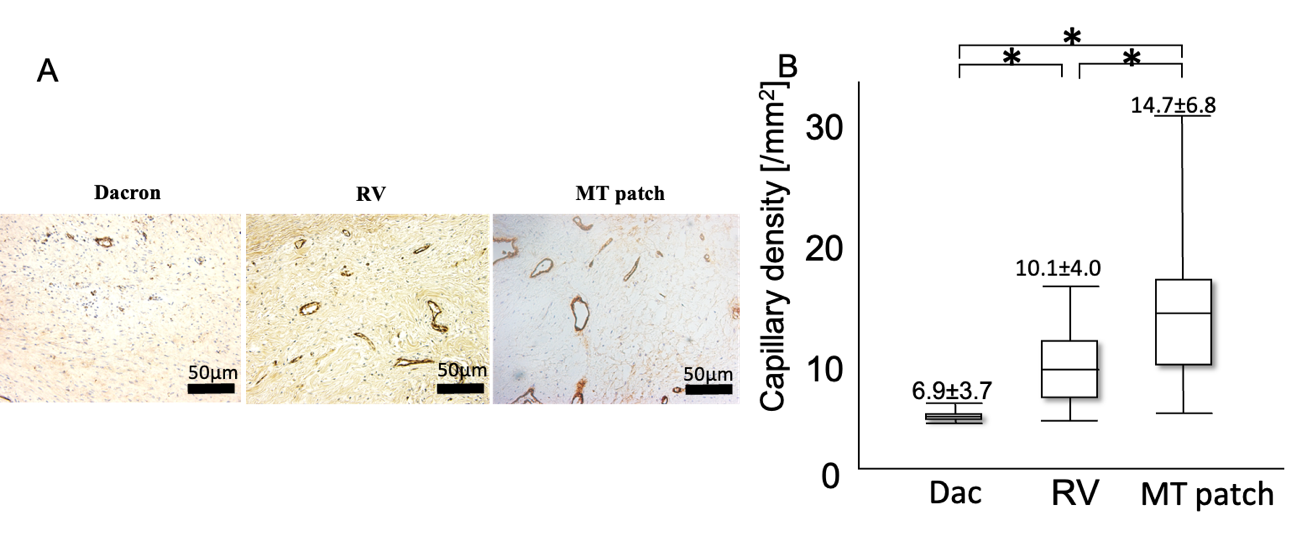


Supplement Figure 2: Capillary density analysis

A: histological results of vWF staining, B: MT patch had significantly higher density of capillary inside of the patch region.


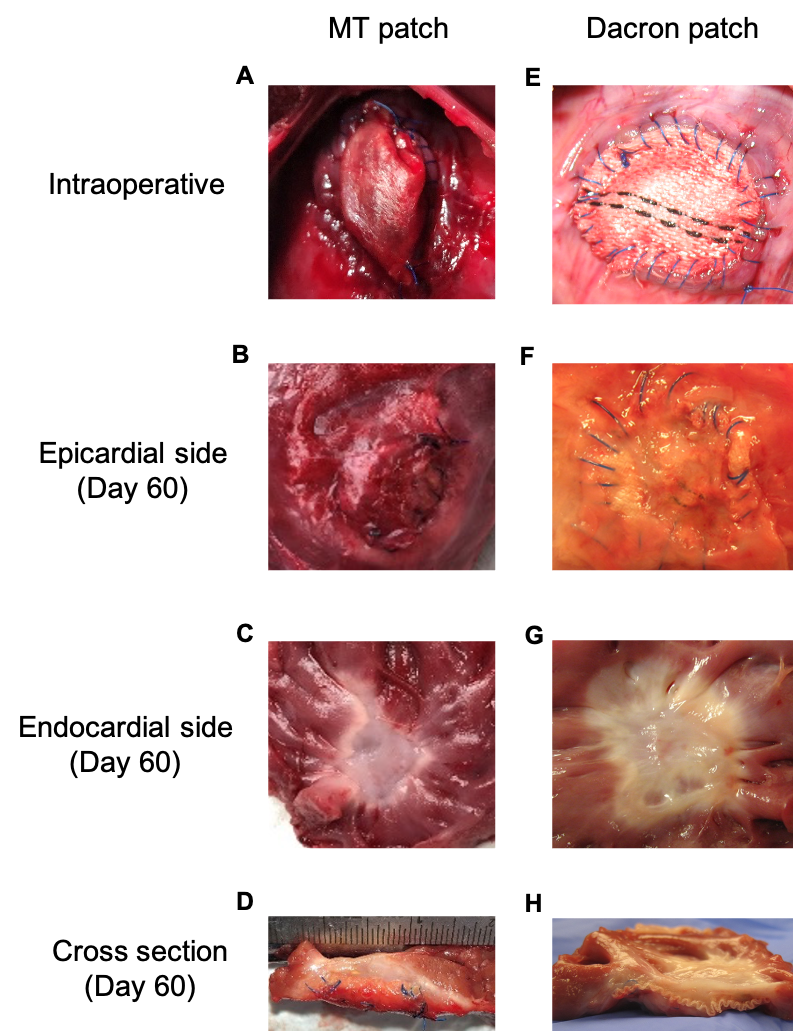


Supplement Figure 3:

No aneurysmal change in the implanted patches were observed in the subjects. The endocardial surface of the patch was covered with well-organized thin white layer tissue(C). In a cross section view, overall the thickness of remodeled tissues in the cardiac patch was equal to that of the normal right ventricle wall(D). The MT patch group had more pink colored tissue in the patch while Dacron group was filled with scar tissue(H).

Supplement Table1

| Supplemental Table 1. Primers and probes for quantitative reverse transcription | | | |
| --- | --- | --- | --- |
| Gene | Primer and Probe | Probe Sequence (5'-3') | |
| GAPDH | Primer forward | CTGCACCACCAACTGCTTAGC | |
|  | Primer reverse | GCCATGCCAGTGAGCTTCC | |
|  | Probe | CCTGGCCAAGGTCATCCATGACCACTTC | |
| SM22α | Primer forward | GCTCCATTTGCTTGAAGACCAT | |
|  | Primer reverse | GTAATGCAGTGTGGCCCTGA | |
|  | Probe | CTCAAAATCACGCCGTTCTTCAGCCA | |
| Vimentin | Primer forward | AGGTGGCAATCTCAATGTCGA | |
|  | Primer reverse | AAATGAGTACCGGAGACAGGTGC | |
|  | Probe | CTCTTCCATTTCCCGCATCTGGCGTT | |
| β-MYH | Primer forward | CTGAAGGACACCCAGATCCA | |
|  | Primer reverse | GTTGATGAGGCTGGTCTGG | |
|  | Probe | ACGCGGTCCGTGCCAATGATGACC | |
| vWF | Primer forward | ATGGAGTACACGGCTTTGCTG | |
|  | Primer reverse | CAGGCACATGCTGTGACACAT | |
|  | Probe | ATGAATGTCCACCTCCTCTTCAGACCGG | |
| FGF-2 | Primer forward | TGTGTGCAAACCGTTATCTTGCTA | |
|  | Primer reverse | CAGTGCCACATACCAACTGGAGTA | |
|  | Probe | CTACAATACTTACCGGTCGAGG | |
| VEGF | Primer forward | GACGTCTACCAGCGCAGCTACT | |
|  | Primer reverse | TTTGATCCGCATAATCTGCATG | |
|  | Probe | TTCCAGGAGTACCCCGATGAGATCGA | |
| β-MYH:beta-myosin heavy chain; FGF-2:fibroblast growth factor-2 | | | |
| GAPDH:Glyceraldehyde-3-phosphate dehydrogenase; SM22α:smooth muscle 22α | | | |
|  | | |  |

Supplement Table 2

| Supplement Table 2. Results of quantitative real-time reverse transcription-polymerase chain reaction | | | |
| --- | --- | --- | --- |
|  | MT Patch | Healthy right ventricle | p |
| SM22α | 1.97±1.26 | 0.04±0.03 | 0.0269* |
| Vimentin | 3.73±2.79 | 0.04±0.02 | 0.0418* |
| β-MYH | 0.0029±0.0041 | 0.0093±0.0034 | 0.0553 |
| vWF | 0.28±0.16 | 0.03±0.01 | 0.0218* |
| FGF-2 | 0.078±0.046 | 0.003±0.001 | 0.0209* |
| VEGF | 0.22±0.14 | 0.04±0.02 | 0.0475* |
| Data are presented as mean (SD). Expressed with the mRNA level/GAPDH.  β-MYH:β-myosin heavy chain; FGF-2:fibroblast growth factor-2, SM22α:smooth muscle 22α | | | |
| VEGF:vascular endothelial growth factor | | |  |
| **p*<0.05 |  |  |  |
